# Antiviral Use in Mild-to-Moderate SARS-CoV-2 Infections during the Omicron Wave in Geriatric Patients

**DOI:** 10.3390/v16060864

**Published:** 2024-05-28

**Authors:** Nadia Exquis, Benjamin Dionisi, Caroline Flora Samer, Victoria Rollason, François Curtin, Dina Zekry, Christophe Graf, Virgnie Prendki, Kuntheavy Ing Lorenzini

**Affiliations:** 1 Division of Clinical Pharmacology and Toxicology, Department of Anesthesiology, Pharmacology, Intensive Care, and Emergency Medicine, Geneva University Hospitals, 1205 Geneva, Switzerland; nadia.exquis@hug.ch (N.E.); caroline.samer@hug.ch (C.F.S.); victoria.rollason@hug.ch (V.R.); francois.curtin@hug.ch (F.C.); 2 Faculty of Medicine, University of Geneva, 1206 Geneva, Switzerland; dina.zekry@hug.ch (D.Z.); christophe.graf@hug.ch (C.G.); virginie.prendki@hug.ch (V.P.); 3 Division of Internal Medicine for the Aged, Department of Rehabilitation and Geriatrics, Geneva University Hospitals, 1205 Geneva, Switzerland; 4 Division of Geriatrics and Rehabilitation, Department of Rehabilitation and Geriatrics, Geneva University Hospitals, 1205 Geneva, Switzerland; 5 Division of Infectious Disease, Geneva University Hospitals, 1205 Geneva, Switzerland

**Keywords:** COVID-19, remdesivir, nirmatrelvir, ritonavir, geriatrics

## Abstract

(1) Background: Geriatric patients are at high risk of complications of Coronavirus disease-2019 (COVID-19) and are good candidates for antiviral drugs. (2) Methods: A retrospective study of electronic health records (EHRs) aiming to describe antiviral (nirmatrelvir and ritonavir (nirmatrelvir/r) or remdesivir) use, drug–drug interactions (DDIs) and adverse drug reactions (ADRs) in elderly patients (75 and over), hospitalized with mild-to-moderate COVID-19 between July 2022 and June 2023. (3) Results: Out of 491 patients (mean age: 86.9 years), 180 (36.7%) received nirmatrelvir/r, 78 (15.9%) received remdesivir, and 233 (47.4%) received no antiviral therapy. No association was found between the choice of antiviral and the demographic or medical data. No serious ADR was observed. Nirmatrelvir/r dosage adjustment was inadequate in 65% of patients with renal impairment. In total, 128 patients (71%) on nirmatrelvir/r had potential pharmacokinetic DDIs, with 43 resulting in a possibly related ADR. In the remdesivir group, pharmacodynamic DDIs were more frequent, with QTc prolongation risk in 56 patients (72%). Only 20 patients underwent follow-up ECG, revealing QTc prolongation in 4. (4) Conclusions: There is an underutilization of antivirals despite their justified indications. Nirmatrelvir/r dosage was rarely adjusted to renal function. Dose adjustments and closer monitoring are needed due to the high risk of drug interactions.

## 1. Introduction

Elderly patients are at high risk of complications from severe acute respiratory syndrome Coronavirus-2 (SARS-CoV-2) infection due to the presence of comorbidities inherent to their age [1,2]. In France, 60% of Coronavirus Disease-2019 (COVID-19)-related deaths involved people aged 80 and over [3]. A retrospective cohort study conducted at Geneva University Hospitals (HUG) that included 235 patients with COVID-19 aged 65 years and over showed 32% mortality [4]. In the second half of 2022, the predominant circulating SARS-CoV-2 variant was Omicron, which appeared to cause less severe disease than previous variants. However, morbidity and mortality in elderly patients were still higher than in the general population [5].

Specific antiviral use is recommended in cases of mild-to-moderate SARS-CoV-2 infection to avoid progression to severe disease [6,7,8]. In a phase 2–3 study, the combination of nirmatrelvir and ritonavir (nirmatrelvir/r) reduced the number of hospitalizations and deaths by day 28 compared with the placebo (relative risk reduction: 89.1%) [9]. Remdesivir, another antiviral drug, stops the replication of SARS-CoV-2 and was the first treatment approved by the US Food and Drug Administration (FDA) against COVID-19 [10]. In a randomized, double-blind, placebo-controlled trial, remdesivir reduced the number of hospitalizations or all-cause deaths at day 28 in outpatients at high risk of complication (hazard ratio 0.13) [11]. This treatment is an attractive alternative for patients who cannot receive nirmatrelvir/r, essentially due to drug–drug interactions (DDIs).

DDIs result from the alteration of the activity of a drug by another drug. They can be classified according to the involved mechanism as either pharmacokinetic (PK) or pharmacodynamic (PD). PK DDIs occur when a drug modifies the absorption, distribution, metabolism, or elimination of another drug, whereas PD DDIs result from additive or antagonistic pharmacological effects [12]. Ritonavir is a perpetrator of many PK DDIs. Indeed, ritonavir is a potent and irreversible cytochrome P450 (CYP) 3A inhibitor, which is used as a PK booster to increase nirmatrelvir plasma concentration. It is also an inhibitor of CYP2D6 and P-glycoprotein (P-gp). CYP inhibition peaks 48 h after the start of treatment and persists for around five days after drug discontinuation [13]. Therefore, it can lead to PK DDIs with medicines whose metabolism is highly dependent on CYP3A. The presence of a drug with a narrow therapeutic index may even contraindicate the use of nirmatrelvir/r. Ritonavir also induces certain CYPs (mainly 2C19) and UDP-glucuronosyltransferases (UGTs). However, induction develops over a few days and is less likely to be significant on short-course treatment. As nirmatrelvir/r reduces complications [9], its use seems appropriate in high-risk patients such as the elderly. However, these patients are often polymedicated, as shown by a 2021 study with an average of 10 drugs prescribed at hospital discharge [14], which increases the risk of DDIs. Moreover, they are often excluded from pre-market drug trials, so approved doses may not be appropriate for adults over 75 [15]. Indeed, certain age-related changes in PK and PD may require caution in medication use. For example, nirmatrelvir/r is partly excreted by the renal route, requiring dose adjustments in case of renal dysfunction.

Unlike nirmatrelvir/r, which has a significant risk of PK DDIs, remdesivir has a lower risk of PK DDIs but a significant risk of PD DDIs due to its potential to prolong the QTc interval. Furthermore, other risk factors may be present in COVID-19 patients, such as hypokalemia [16] or the presence of other drugs known to prolong the QTc interval [17].

At HUG and in line with the current international and national guidelines, nirmatrelvir/r is used as a form of early treatment to prevent progression to severe disease in mild-to-moderate COVID-19, mainly in high-risk patients (immunosuppression, an oncological or hematological disease, solid organ transplantation, >75 years or >60 years, without a full vaccination schedule or with high-risk factors) [7]. In cases of contraindication, remdesivir can be prescribed instead. We aimed to assess the frequency and adequacy of the use of these antiviral drugs in elderly patients in a real-life setting. As our study was purely descriptive, no a priori hypothesis was formulated.

## 2. Materials and Methods

### 2.1. Study Design, Setting, and Population

We conducted a monocentric retrospective observational study within the HUG Department of Rehabilitation and Geriatrics at Trois-Chêne Hospital, a 250-bed secondary hospital dedicated to the care of elderly patients. Through the automated extraction of patient’s electronic health records (EHRs), we identified patients hospitalized at Trois-Chêne Hospital from 1 July 2022 to 30 June 2023 with a positive SARS-CoV-2 test. The EHRs were reviewed to determine whether they met the inclusion criteria, i.e., inpatients aged ≥75 with a mild-to-moderate SARS-CoV-2 infection diagnosed by PCR testing. Patients were excluded in the following cases: severe COVID-19, false positive test, positive test at the end of a long-standing infection, or signed or orally expressed refusal to use their clinical data (general consent). According to the World Health Organization’s (WHO’s) definition of a post-COVID-19 condition, we considered a long-term infection up to 3 months after the positive test [18]. The WHO defines severe COVID-19 as clinical signs of pneumonia with one of the following criteria: respiratory rate > 30 breaths/min, severe respiratory distress, or SpO2 < 90% on room air [19].

### 2.2. Outcomes

The primary outcome was to assess the frequency of use of nirmatrelvir/r and remdesivir. Secondary outcomes included a description of DDIs with current comedications and adverse drug reactions (ADRs), as well as whether antivirals were used as indicated in the summary of product characteristics (SPCs). We considered the WHO definition of ADR as “a response to a medicine which is noxious and unintended, and which occurs at doses normally used in man” [20]. We also collected relevant medical data (comorbidities, current medications, SARS-CoV-2 vaccination status) and hospitalization data (reasons for early antiviral treatment discontinuation, duration of acute care hospitalization, transfer to intensive care unit (ICU) or death).

### 2.3. Data Source and Variables

The following data were collected from the EHR: demographics, comorbidities, Charlson comorbidity index (CCI), Functional Independence Measure (FIM) [21] and Clinical Frailty Scale [22], SARS-CoV-2 vaccinal status, renal function (Cockcroft formula), QTc at baseline, the reason for hospitalization, date of positive SARS-CoV-2 test and date of antiviral introduction, source of SARS-CoV-2 infection (nosocomial, i.e., any infection that occurs at least 48 h after admission [23] or that is community-acquired), treatments received during hospitalization, and issue of hospitalization including complications (heart failure, respiratory failure, pulmonary embolism, bacterial superinfection, other (Table S1)). Comorbidities were classified into the following ten categories: cardiovascular, pulmonary, metabolic, hepatic, neuropsychiatric, renal, geriatric, neoplastic, musculoskeletal, and others (Table S2). The risk of interaction between antiviral drugs and current treatment was assessed using the University of Liverpool’s COVID-19 interactions resource (www.COVID19-druginteractions.org) [24]. This resource classifies potential DDIs into red flags (the drug combination should not be co-administered), amber flags (potential clinical DDIs manageable), yellow flags (weak clinical relevance for DDIs), and green flags (no DDI).

### 2.4. Statistics

Primary and secondary outcomes were reported using descriptive statistics. For analysis, IBM SPSS 25 software (IBM Inc., Chicago, IL, USA) was used. Frequencies and percentages were generated for qualitative variables, while means with confidence intervals were used for quantitative variables. When two groups were analyzed, Student’s *t*-tests for continuous variables and Fisher’s exact tests for categorical variables were used. Chi-square analyses for categorical variables and ANOVA analyses were used for continuous variables when more than two groups were analyzed. A statistical significance level of *p* < 0.05 was defined to establish a statistical difference.

### 2.5. Ethics Approval

This study was conducted with ethical standards in accordance with the Declaration of Helsinki. Ethical approval for the study was obtained from the local ethics committee (local study number: GE-CCER 2023-00943). Some patients signed a general consent agreement for the reuse of their data for medical research. For other cases, an exception was granted by the local ethics committee. Patients who had signed or expressed an oral refusal to the clinical use of their data were excluded.

## 3. Results

**Figure 1:**
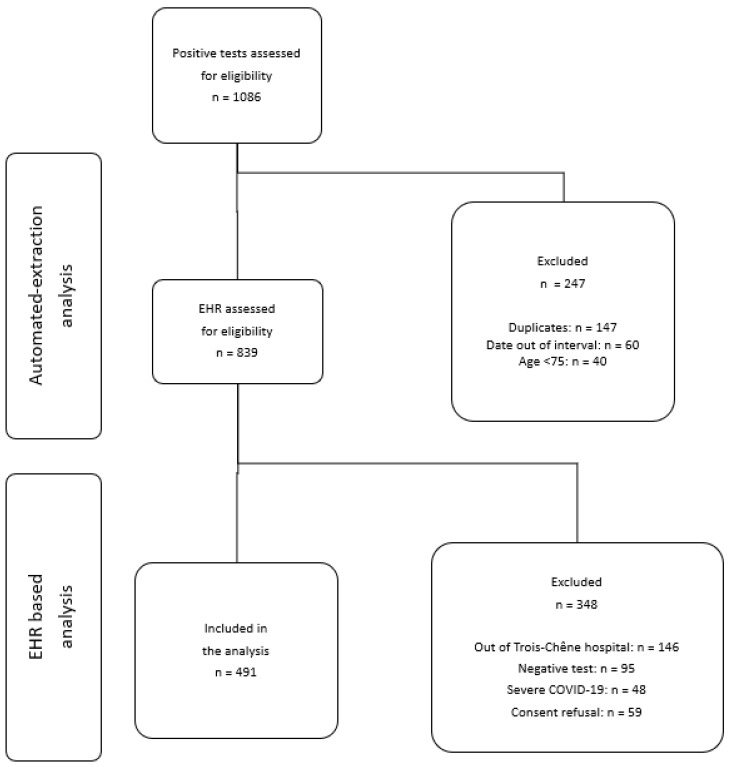
Study flow chart.

A total of 1086 positive SARS-CoV-2 tests were identified in patients hospitalized during the study period. We excluded 247 cases because they did not meet the inclusion criteria. After reviewing the EHRs of the remaining 839 cases, we excluded a further 348 for various reasons described in Figure 1. Finally, 491 patients were included in the analysis (Figure 1). Patients were divided into the following three groups: 180 patients (36.7%) received nirmatrelvir/r, 78 (15.9%) received remdesivir, and 233 (47%) received no antiviral treatment. No patient received both antiviral drugs.

The patients’ mean age was 86.9 years (range 75–102), and 61.9% were females (Table 1). Most (96.9%) patients lived at home with or without care (71.3% and 25.7%, respectively), and only 3.1% lived in long-term care facilities. The mean CCI was 6.7, meaning that the estimated 10-year survival was 2%, and the mean FIM was 81/126. Patients had an average of 10 comorbidities, mainly cardiovascular (hypertension, atrial fibrillation, ischemic cardiopathy, and heart failure) and geriatric (risk of falls, cognitive impairment, protein-energy malnutrition). They received an average of 12 medications per day. The most frequent causes of hospital admission were falls, respiratory problems (e.g., infectious origin or COPD), and deteriorating general conditions. Comparing patients who received no antiviral treatment with those who received nirmatrelvir/r or remdesivir, we found no association between demographics, comorbidities, and the antiviral prescription.

The origin of SARS-CoV-2 infection was nosocomial in 255 patients (51.9%). Cough, fever, asthenia, and respiratory problems were the most frequent symptoms. Cough and fever were more common in both antiviral groups than in the no-treatment group (*p* = 0.048 for cough in the nirmatrelvir/r group, *p* = 0.013 for cough in the remdesivir group, and *p* = 0.003 for fever in the remdesivir group). With regard to the SARS-CoV-2 vaccination status, 348 patients (70.9%) received at least one dose of the vaccine, and 265 completed the recommended vaccination schedule. Sixty patients (12.2%) had received no dose (Table 2). Information was missing for 83 patients (16.9%). No association was found between the vaccination status and choice of antiviral therapy.

With regard to antiviral treatment, more than half the patients (53%) received antiviral treatment; as previously mentioned, 180 patients (36.7%) received nirmatrelvir/r, and 78 (15.9%) received remdesivir (Table 3). In most cases, the reason for not prescribing antiviral treatment was not explicitly documented in the EHR. The mean time from the SARS-CoV-2 positive test to the first dose was longer for remdesivir (1.67 vs. 1.21 days, *p* = 0.046) (Table 3). Of the 258 patients treated, antiviral therapy was discontinued prematurely in 20 patients (17 in the nirmatrelvir/r group and 3 in the remdesivir group). The reasons for this were as follows: DDIs (n = 8), refusal of treatment (n = 6), death (n = 2), ADR (n = 2, only for remdesivir: nausea and skin rash), decreased renal function (n = 1) and loss of ability to swallow (n = 1).

Renal function was determined in 486 patients. Over half of the patients (n = 299, 60.9%) had KDIGO stage 3 chronic kidney disease (CKD) (creatinine clearance 30–59 mL/min), while 123 patients (25.1%) had severe-to-end-stage CKD (creatinine clearance 0–29 mL/min). There was no difference between the treatment groups in the proportions of CKD stages. The dose of nirmatrelvir/r was adapted to renal function according to the SPC recommendation for only 31% of patients with moderate-to-end-stage renal disease (Table 4).

In the nirmatrelvir/r group, patients (n = 180) received a mean number of 11 concomitant drugs (Table 3). We detected 247 potential PK DDIs in 128 patients (71%). As shown in Table 5, most of these involved cardiovascular drugs (torasemide, valsartan, and amlodipine), for which changes in blood pressure (both hypotension and hypertension) (yellow or amber flags) were expected as a consequence. Other frequently involved classes were psychotropic drugs and opioids. Quetiapine is contraindicated with strong CYP3A4 inhibitors such as ritonavir (red flag) [25], and morphine efficacy may be reduced by ritonavir (induction of UGT2B7) (amber flag) [25]. In two cases, we detected a potential DDI with nirmatrelvir as the victim drug as follows: the concomitant use of metamizole could lead to a decrease in nirmatrelvir exposure via the induction of CYP3A4. Of these 128 patients, we suspected that DDIs eventually caused an ADR in 43 patients. The ADRs observed were blood pressure changes with antihypertensive agents, tachycardia, confusion or sedation with antipsychotics and antidepressants, and loss of efficacy or confusion with opioids.

**Figure 2:**
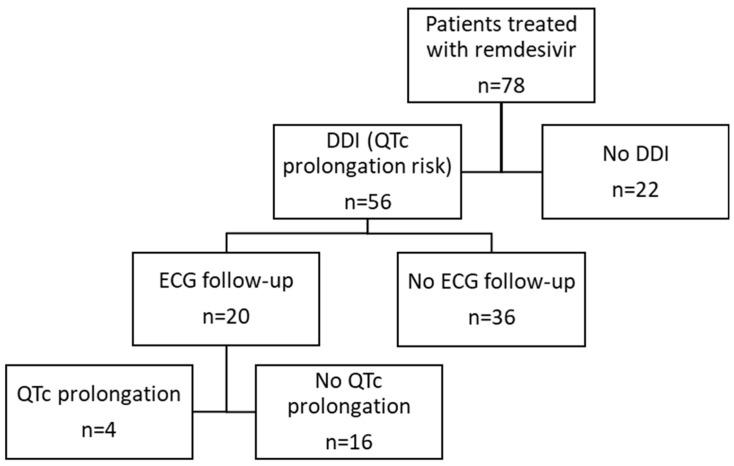
Follow-up ECG in the remdesivir group in patients with QTc prolongation risk.

In the remdesivir group, a theoretical PK DDI was detected in 4 patients, but no clinical impact was observed. PD DDIs leading to a risk of QTc prolongation were the most frequent (n = 56 patients, 72%) and mainly concerned metoprolol, mirtazapine, and amlodipine. The mean QTc interval at the baseline, calculated according to the Bazett formula, was 453 ms in our global population. It was longer in the non-treated group (457 ms) and the remdesivir group (451 ms) than in the nirmatrelvir/r group (449 ms) (*p* = 0.016) (Table 3). Of the 56 patients at risk of a prolonged QTc interval due to DDI, a follow-up electrocardiogram (ECG) during remdesivir therapy was performed in only 20 patients. QTc prolongation was observed in four of these patients (Figure 2).

On average, patients remained in the hospital for 22.2 days, with no statistically significant difference between groups (*p* = 0.09). Complications were observed in 222 patients (45.2%), with the most frequent being heart failure (n = 80), bacterial superinfection (n = 70), and respiratory failure (n = 37). No patient was transferred to an intensive care unit, and only eight patients were transferred to the intermediate care unit. Most patients (n = 276, 55.6%) returned home at the end of hospitalization, while 53 (10.8%) were transferred to another care unit (e.g., rehabilitation unit, psychiatric unit); no differences were observed between groups. A total of 26 patients (5.3%) died during hospitalization, but only six deaths were undeniably linked to SARS-CoV-2 infection, according to the death certificate.

## 4. Discussion

In our study of an elderly population hospitalized with mild-to-moderate SARS-CoV-2 infection, we found that around half the patients received antiviral treatment. The patients were representative of the Swiss geriatric population, with the majority of women living at home with or without care [26]. The mean age of the patients was 86.9 years old, and the oldest was 102; however, few data are available on the use of antivirals against SARS-CoV-2 in a very elderly population. In the phase 2–3 study of nirmatrelvir/r, the mean age was 46 years (range 18–88) [9], while in the remdesivir study, it was 50 years (SD 15) [11]. To the best of our knowledge, only one study has specifically examined the tolerability of nirmatrelvir/r in patients over 80 years of age (16 patients aged 80–89, five patients over 90) [27]. Other studies involving elderly patients included only a limited number of patients over 80 years of age. For example, in a retrospective cohort study including 13,861 patients treated with nirmatrelvir/r, 37.5% of participants were aged ≥80 years, 21.1% were 75–79 years old, and 21.3% were 70–74 years [28]. The authors showed the most significantly reduced risk of hospital admission or death from COVID-19 in patients aged >70 years. With regard to remdesivir, a study focused on hospitalized patients older than 80 (n = 140) and showed a 15.7% reduction in 30-day all-cause mortality compared with no treatment [29].

Our patients had a mean number of 10 comorbidities and 12 daily drugs, which is comparable to previous studies [14]. The SARS-CoV-2 vaccination rate was in line with that of the Swiss population, which was 69.8% in November 2023 (at least one vaccine dose) [30].

The other half of patients who did not receive any antiviral therapy had fewer symptoms of COVID-19 than treated patients. Nevertheless, national and international guidelines [6,7,8] recommend antiviral target therapy for mild COVID-19 in people at high risk of complications, irrespective of their vaccination status and comorbidities. Geriatric patients are generally included in this group. The use of remdesivir may be preferred in the case of hepatic impairment or clinically significant drug interactions [7]. Antivirals were underused in our population, although the underlying reasons for this are unclear. One hypothesis could be concern about the economic costs of antivirals. However, according to a systematic literature review of studies conducted during periods of Omicron predominance, antivirals are effective at reducing healthcare resource use by avoiding hospital admissions compared to no treatment in outpatients in the USA [31]. A US study showed that the risk of post-acute hospitalization at 180 days was lower in patients at risk of progression to severe COVID-19 treated with nirmatrelvir/r than in those who received no treatment [32]. However, a cost-effectiveness analysis conducted in Spain for nirmatrelvir/r in a simulated cohort of 100,000 outpatients aged over 65 with mild-to-moderate COVID-19 and risk factors for complications concluded that this treatment would not be cost-effective [33]. Further pharmacoeconomic studies could help determine the cost-effectiveness of antivirals against COVID-19, especially in the very elderly population.

The dose of nirmatrelvir/r has not been systematically adjusted for renal function in our study, even though clear recommended regimens are proposed [25]. While nirmatrelvir is rapidly metabolized by CYP3A when administered alone, in combination with ritonavir, 50% is excreted as unchanged form by the kidneys. As a result, nirmatrelvir exposure may be increased in patients with renal impairment, although the literature provides little information on its use in renal impairment [34].

The longer delay between the SARS-CoV-2 positive test and the first dose of remdesivir may reflect the time needed to assess the DDIs involving nirmatrelvir/r before choosing remdesivir, which is the second-line therapy. Another explanation could be the need for intravenous administration.

Only two ADRs were specifically documented in the EHR as being related to antivirals. However, antivirals are perpetrators of numerous potential DDIs. DDIs may result in ADRs, as shown by several research groups. For example, a retrospective pharmacovigilance study reported that one-third of patients exposed to a potential DDI presented a serious ADR [35]. In our study, we detected a PK DDI in 128 patients (71%) of the nirmatrelvir/r group. In 43 of these, an ADR occurred, probably due to DDIs. DDIs mostly involved CYP3A (Table 5), which is not surprising since CYP3A metabolizes around 50% of the drugs [36]. The inhibition of CYP3A by ritonavir can lead to the increased plasma concentration of substrates, thus enhancing the risk of ADR. DDIs involving CYP2C9 or UGT induction, which can lead to a loss of efficacy of substrates, were less frequent. Furthermore, a few “red-flag” DDIs and ADRs were observed with quetiapine. ADRs resulting from DDIs are considered avoidable ADRs. Several methods for evaluating ADR preventability have been used in the literature. Generally, prescribing errors, inadequate monitoring, and DDIs are considered the main causes of avoidable ADRs [37,38,39]. As a result, we consider that all ADRs possibly linked to a DDI are preventable in our study. Checking DDIs and adjusting doses where necessary could help reduce these ADRs. The role of clinical pharmacologists in antiviral selection, dose adjustment, DDI assessment and management, and patient monitoring during therapy should be emphasized.

With regard to remdesivir, we mainly found PD DDIs with a risk of QTc prolongation. At the baseline, the QTc interval was longer in patients in the non-treated group. We hypothesized that QTc prolongation could have been a barrier to the prescription of remdesivir or that this difference was simply due to chance. In addition, we observed that follow-up ECGs were not systematically performed even when a DDI was identified. Education for prescribers should be reinforced to optimize this follow-up.

The mostly positive issue of hospitalization with no ICU transfer and only six deaths linked to COVID-19 could be due to our exclusion criteria, as severe cases were not included in our study.

Our single-center retrospective EHR-based study has certain limitations. Firstly, we cannot exclude that relevant items were not compiled in the EHR during hospitalization. In addition, only concomitant drugs were identified, and we did not describe changes in treatment (e.g., temporary discontinuation) that took place in anticipation of antiviral treatment, which may have led to an underestimation of red-flag DDIs.

## 5. Conclusions

In geriatric patients hospitalized with mild-to-moderate COVID-19, nirmatrelvir/r was used more often than remdesivir, but there was no association between the choice of an antiviral, patient medical history, and SARS-CoV-2 infection characteristics. Although guidelines recommend antiviral therapy in our population, we found the underutilization of these drugs. In a future study, barriers to prescribing should be assessed in further detail. A survey of prescribers could help determine reasons representing barriers to antiviral prescribing, such as their high cost, lack of data in very elderly patients, or fear of DDIs. In addition, the nirmatrelvir/r dose was infrequently adjusted to renal function. Nirmatrelvir/r and remdesivir were associated with a higher risk of PK and PD DDIs, respectively, leading to clinically significant ADRs that may require closer monitoring during treatment. Monitoring DDI and adjusting drug doses accordingly could reduce the incidence of ADRs.

## Figures and Tables

**Table 1:** Patients’ demographics and medical conditions at baseline.

Male, n (%)	187 (38.1%)
Female, n (%)	304 (61.9%)
Age, mean (SD)	86.9 (0.3)
Lifestyle before hospitalization	
Living at home with a carer, n (%)	350 (71.3%)
Living at home without a carer, n (%)	126 (25.7%)
Living in long-term care facilities, n (%)	15 (3.1%)
Charlson Comorbidity Index, mean (SD)	6.7 (0.1)
Functional Independence Measure, mean (SD)	80.7 (1.2)
Number of comorbidities, mean (SD)	10 (3.9)
Most frequent type of comorbidities	
Arterial hypertension, n	353
Atrial fibrillation, n	181
Ischemic cardiopathy and heart failure, n	179
Risk of falling, n	212
Cognitive impairment, n	182
Protein-energy malnutrition, n	177
Most frequent causes of hospital admission	
Falls, n (%)	90 (18.3%)
Respiratory problems, n (%)	57 (11.6%)
Decline in general condition, n (%)	56 (11.4%)
Length of stay, mean (SD)	22.2 days (0.7)
Death, n (%)	26 (5.3%)

**Table 2:** SARS-CoV-2 vaccination status and infection characteristics.

Vaccinal Status	
At least one dose of vaccine, n (%)	348 (70.9%)
Complete vaccination schedule, n (%)	265 (64.9%)
Not vaccinated, n (%)	60 (12.2%)
Missing information, n (%)	83 (16.9%)
Nosocomial infection, n (%)	255 (51.9%)
Community-acquired infection, n (%)	236 (48.1%)

**Table 3:** Antiviral treatment characteristics.

	Nirmatrelvir/r	Remdesivir	No Treatment	All Groups
n (%)	180 (36.7%)	78 (15.9%)	233 (47%)	491 (100%)
Delay between positive test and first dose, mean (SD)	1.21 days (1.16)	1.67 days (1.87)	-	1.34 days (1.42)
QTc at baseline, mean (SD)	449 ms (26.8)	451 ms (30.7)	457 ms (32)	453 ms (1.4)
QTc at baseline (females), mean (SD)	448 ms (27)	455 ms (31.8)	457 ms (31.9)	453 ms (30.3)
QTc at baseline (males), mean (SD)	450 ms (26.6)	446 ms (28.9)	457 ms (32.3)	453 ms (29.9)
Number of daily drugs, mean (SD)	11 (4.2)	11 (3.8)	13 (5.3)	12 (4.8)

**Table 4:** Nirmatrelvir’s daily dose distribution based on creatinine clearance.

	CrCl * <15	CrCl * 15–29	CrCl * 30–59	CrCl * 60–89	CrCl* >90
300 mg B.D.	0	3	69	18	3
150 mg B.D.	0	29	36	0	0
Loading dose of 300 mg, and then 150 mg O.D.	3	11	5	0	0
150 mg O.D.	1	1	0	0	0
Dose adapted to renal function (%)	31%			100%	100%

CrCl: creatinine clearance; *: mL/min; grey: correct dose according to renal function.

**Table 5:** Drugs most frequently involved in potential pharmacokinetic DDIs with nirmatrelvir/r.

Drugs	Level of Risk	Mechanism of DDI	Plasma Concentration Change
Torasemide (n = 43)	Yellow flag	CYP2C9 induction	Decrease
Amlodipine (n = 30)	Amber flag	CYP3A4 inhibition	Increase
Morphine (n = 15)	Amber flag	UGT induction	Decrease
Valsartan (n = 11)	Amber flag	OATP1B1 and MRP2 inhibition	Increase
Mirtazapine (n = 10)	Yellow flag	CYP2D6 and CYP3A4 inhibition	Increase
Atorvastatin (n = 8)	Amber flag	CYP3A4 inhibition	Increase
Venlafaxine (n = 8)	Yellow flag	CYP2D6 inhibition	Increase
Acenocoumarol (n = 7)	Amber flag	CYP2C9 induction	Decreased
Quetiapine (n = 7)	Red flag	CYP3A4 inhibition	Increased
Buprenorphine (n = 6)	Yellow flag	CYP3A4 inhibition	Increased
Irbesartan (n = 6)	Yellow flag	CYP2C9 induction	Decreased
Brinzolamide (n = 5)	Amber flag	CYP3A4 inhibition	Increased
Ezetimibe (n = 5)	Yellow flag	UGT induction	Decreased
Losartan (n = 5)	Yellow flag	CYP2C9 induction	Decreased
Tamsulosine (n = 5)	Amber flag	CYP3A4 inhibition	Increased
Zolpidem (n = 5)	Amber flag	CYP3A4 inhibition	Increased

## Data Availability

The datasets used and/or analyzed during the current study are available from the corresponding author on reasonable request due to data privacy concerns.

## Supplementary Materials

The following supporting information can be downloaded at: https://www.mdpi.com/article/10.3390/v16060864/s1, Table S1: Other complications of hospitalization sought in the EHR; Table S2: Comorbidities sought in the EHR.
