# Supplementary material for: Antiviral Use in Mild-to-Moderate SARS-CoV-2 Infections during the Omicron Wave in Geriatric Patients
Source: Viruses. 2024 May 28;16(6):864. doi: 10.3390/v16060864 (PMC11209592; doi:10.3390/v16060864)
Supplement: Supplementary file 1 [file viruses-16-00864-s001.zip › viruses-3022120-Supplementary materials.pdf]

Supplementary materials

Table S1. Other complications of hospitalization sought in the EHR

NSTEMI
Arterial pressure variation
Stroke
Cerebrovascular bleeding
Digestive bleeding
Hepatobiliary anomalies
Pancreatitis
Blood pH variations
SIADH
Diabetic decompensation
Alteration of blood cells count
Extrapyramidal symptoms
Psychiatric decompensation
Anxiety
Delirium
Falls
Arthritis exacerbation
Peptic ulcer disease
Uncontrolled pain
Opioids intoxication
Pleural effusion
Articular effusion
Renal insufficiency
Adrenal insufficiency
Mycosis
Zona reactivation
ORL disease

Table S2. Comorbidities sought in the EHR

Cardiovascular comorbidities	Arterial hypertension
	Arterial hypotension
	Orthostatic hypotension
	Atrial fibrillation
	Cardiac insufficiency
	Arrhythmia
	Venous thrombosis
	Stroke
	Myocardial infarct
Pulmonary comorbidities	COPD
	Asthma
	Allergy
	Pulmonary hypertension
	Fibrosis
	Pulmonary embolism
Metabolic comorbidities	Diabetes mellitus (type 1 and type 2)
	Dysthyroid
	Dyslipidemia
Hepatic comorbidities	Acute hepatocellular insufficiency
	Chronic hepatocellular insufficiency or cirrhosis
	Viral hepatitis
Neuropsychiatric comorbidities	Schizophrenia
	Depression/anxiety
	Bipolar disorder
	Insomnia
	Dementia
	Epilepsy
	Parkinson disease
Renal comorbidities	Acute renal failure
	Chronic renal failure
	Benign prostatic hyperplasia
	Urinary incontinence
Geriatric comorbidities	Hearing dysfunction
	Impaired mobility
	Malnutrition
	Decline in general condition
	Falls
	Delirium
	Cognitive impairment
	Force deficit
Neoplastic disease	Active cancer/lymphoma
	History of cancer
Musculoskeletal comorbidities	Chronic pain
	Low back pain
	Arthrosis
	Arthritis
	Gout
	Hernia
	Osteoporosis
Other comorbidities	Cardiac valvulopathy
	QT interval prolongation

	Arterial aneurysm
	Arterial stenosis
	Arteritis
	Aphasia
	Anemia
	Cytopenia
	Monoclonal gammopathy, MGUS
	Adrenal insufficiency
	Hyperparathyroidism, hypoparathyroidism
	Pancreatic insufficiency
	Gastroesophageal reflux disease, gastritis, peptic ulcer disease
	Dysphagia
	Gastrointestinal bleeding
	Hepatic abscess
	Hepatic steatosis
	Obesity
	Sleep apnea
	Restless legs syndrome
	Alcoholism
	Polyneuropathy
	Neuralgia
	Multiple sclerosis
	Essential tremor
	Polymyalgia rheumatica
	Urinary retention
	Ophthalmic disease
	Cutaneous disease
	Fibromyalgia
	Vertigo
	Ménière’s disease
	Smoking

6
